# Use of response surface methodology for development of new microwell-based spectrophotometric method for determination of atrovastatin calcium in tablets

**DOI:** 10.1186/1752-153X-6-134

**Published:** 2012-11-12

**Authors:** Tanveer A Wani, Ajaz Ahmad, Seema Zargar, Nasr Y Khalil, Ibrahim A Darwish

**Affiliations:** 1 Department of Pharmaceutical Chemistry, College of Pharmacy, King Saud University, P.O. Box 2457, Riyadh, 11451, Saudi Arabia; 2 Department of Clinical Pharmacy, College of Pharmacy, King Saud University, P.O. Box 2457, Riyadh, 11451, Saudi Arabia; 3 Department of Biochemistry, College of Science, King Saud University, P.O. Box 22452, Riyadh, 11211, Saudi Arabia

**Keywords:** Response surface methodology, Atorvastatin, Validation, Optimization, Tablets

## Abstract

**Background:**

Response surface methodology by Box–Behnken design employing the multivariate approach enables substantial improvement in the method development using fewer experiments, without wastage of large volumes of organic solvents, which leads to high analysis cost. This methodology has not been employed for development of a method for analysis of atorvastatin calcium (ATR-Ca).

**Results:**

The present research study describes the use of in optimization and validation of a new microwell-based UV-Visible spectrophotometric method of for determination of ATR-Ca in its tablets. By the use of quadratic regression analysis, equations were developed to describe the behavior of the response as simultaneous functions of the selected independent variables. Accordingly, the optimum conditions were determined which included concentration of 2,3-dichloro-5,6-dicyano-1,4-benzoquinone (DDQ), time of reaction and temperature. The absorbance of the colored-CT complex was measured at 460 nm by microwell-plate absorbance reader. The method was validated, in accordance with ICH guidelines for accuracy, precision, selectivity and linearity (*r*² = 0.9993) over the concentration range of 20–200 μg/ml. The assay was successfully applied to the analysis of ATR-Ca in its pharmaceutical dosage forms with good accuracy and precision.

**Conclusion:**

The assay described herein has great practical value in the routine analysis of ATR-Ca in quality control laboratories, as it has high throughput property, consumes minimum volume of organic solvent thus it offers the reduction in the exposures of the analysts to the toxic effects of organic solvents, environmentally friendly "Green" approach) and reduction in the analysis cost by 50-fold.

## Background

**Figure 1:**
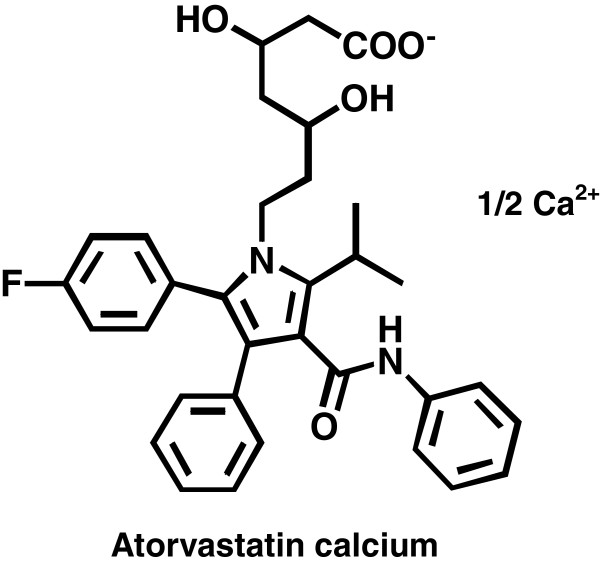
The chemical structure of Atorvastatin Calcium (ATR-Ca).

Atorvastatin calcium (ATR-Ca); [(R-(R*,R*)]-2-(4-fluorophenyl)-β,δ,dihydroxy-5-(1-methylethyl)-3-phenyl-4-[(phenyl-amino)-carbonyl]-1H-pyrrole-1-heptanoic acid calcium salt (Figure 1), is a second generation synthetic 3-hydroxy-3-methylglutaryl-coenzyme A (HMG-CoA) reductase inhibitor [1]. It exerts its action by specifically inhibiting the HMG-CoA reductase, the enzyme that catalyzes the conversion of HMG-CoA to mevolanate, which is the early rate-limiting step in the biosynthesis of cholesterol in the body. ATR-Ca decreases the amount of LDL-cholesterol in the blood and also reduces blood levels of triglycerides and slightly increases levels of HDL-cholesterol. ATR-Ca is the most efficient and frequently prescribed drug for the treatment of hypercholesterolaemia [2].

Response surface methodology (RSM) is a statistical technique used for the development and optimization of complex processes [3,4]. It was selected and used to find the optimum spectrophotometric conditions for analysis of atorvastatin. The technique has several advantages over conventional experimental or optimization methods in which one variable at a time is used. RSM provides a large amount of information and is more economical approach because a small number of experiments are performed for monitoring the interaction of the independent variables on the response. In conventional optimization, the increase in the number of experiments necessary to carry out the research, leads to an increase in time and expenses as well as an increase in the utilization of reagents and materials for experiments. The equation of the model easily clarifies the effects for binary combinations of the independent variables. Many types of response surface designs are used for optimization like Central composite, Doehlert, and Box–Behnken. Box–Behnken design is preferable to the Central composite and Doehlert designs because it requires fewer test runs and is rotatable. A design is rotatable only when the experiments are roughly situated on a (hyper) sphere. By adequate selection of the number of centre points, it is possible to arrange that the precision of the response of a predicted design is similar over the whole domain. Such a design is said to have uniform precision.

Box–Behnken design is advantageous because it does not contain any points at the extremes of the cubic region created by the two-level factorial level combinations [3-5]. In the present investigation the Box Behnken design was selected and used to optimize, validate and analyze the atorvastatin spectrophotometrically, because the design provides three levels for each factor and requires fewer runs in the three-factor case than Central composite and Doehlert design.

In general, UV-visible spectrophotometry is the most widely used technique in pharmaceutical analysis because of its inherent simplicity and wide availability in most quality control laboratories [6-10]. However, the UV-visible spectrophotometric methods that have been reported for determination of ATR-Ca in its pharmaceutical formulations [11-17] suffer from major drawbacks. These drawbacks include decreased selectivity due to measuring the native light absorption of ATR-Ca in the blue-shifted ultraviolet region, which might be subjected to interferences [13], employment of multiple-steps of non-selective oxidation reactions [14-16], and tedious liquid-liquid extraction procedures using large volumes of organic solvents in the methods based on formation of ion-pair associates [17]. Microwell based automated spectrophotometric method for determination of ATR-Ca using colored charge-transfer (CT) complex has been developed as alternative for the conventional spectrophotometric technique [18]. Moreover, the conventional methods suffer from the consumption of large volumes of organic solvents, which leads to high analysis cost, and more importantly, the incidence of exposure of the analysts to the toxic effects of the organic solvents [19-23]. These days, there is an urge in scientific community for the improvement in existing analytical methods in such a way that the developed approach includes both the positive traits (selectivity and sensitivity) of the traditional methods as well as the environmentally friendly "Green" approach, in which the amount of hazardous reagents used and chemical waste generated during analysis should be minimal.

Considering the variability of spectrophotometric conditions used in analysis of atorvastatin by CT reaction, need for cost reductions and need for environmentally friendly "Green" approach in routine pharmaceutical analysis, the objective of this research was to optimize and validate CT based reaction of ATR-Ca, and its employment in the optimization of simple and reliable automated microwell based spectrophotometric assay with high throughput analysis. A multivariate approach was adopted as an analytical tool to study the effect of different parameters and to improve spectrophotometric conditions for the analysis of ATR-Ca.

## Methods

### Apparatus

Microwell-plate absorbance reader (ELx 808, Bio-Tek Instruments Inc. Winooski, USA) was used for all the measurements in 96-microwell plates. 96-Microwell plates were a product of Corning/Costar Inc. (Cambridge, USA). Finnpipette adjustable 8–channel-pipette was obtained from Sigma Chemical Co. (St. Louis, MO, USA).

### Chemicals and dosage forms

ATR-Ca was obtained from Pfizer Inc. (New York, USA). DDQ was obtained from (Merck, Germany). Lipitor tablets (Parke Davis, Germany) and Lipicure-10 tablets (INTAS Pharmaceuticals, India) labeled to contain 10 mg ATR-Ca were obtained from the local market and from India.

### Preparation of standard and tablet solutions

#### Preparation of stock standard solutions

Into a 5-ml calibrated flask, 10 mg of ATR-Ca was accurately weighed, dissolved in 2 ml methanol, and completed to volume with the same solvent. This stock solution was diluted with methanol to obtain the suitable concentrations that lie in the linear range of the assay.

#### Preparation of DDQ solutions

Into a 5-ml calibrated flask, 8 mg of DDQ was accurately weighed, dissolved in 2 ml methanol, and completed to volume with the same solvent.

#### Preparation of tablet sample solutions

Twenty tablets were weighed and finely powdered. A quantity of the powder equivalent to 20 mg of ATR-Ca was transferred into a 10-ml calibrated flask, dissolved in 4 ml methanol, swirled and sonicated for 5 min, completed to volume with the methanol, shaken well for 15 min, and filtered. The first portion of the filtrate was rejected, and a measured volume of the filtrate was diluted quantitatively with methanol to yield the suitable concentrations that lie in the linear range of the assay.

### Analytical procedure

Accurately measured aliquots (100 μl) of the standard or sample solution containing varying amounts of ATR-Ca (20–200 μg) were transferred into wells of 96-microwell assay plates. One hundred microliters of DDQ solution (0.16%, w/v) was added, and the reaction was allowed to proceed at (31 ± 1°C) for 3.5 min. The absorbances of the resulting solutions were measured at 460 nm by the microwell-plate reader. Blank wells were treated similarly except 100 μl of methanol was used instead of sample, and the absorbances of the blank wells were subtracted from those of the other wells.

### Optimization of experimental conditions

#### Box–Behnken experimental design

A response surface statistical experimental design was used to optimize the concentration of DDQ, time, and temperature. This design was based on a 3^3^ factorial design, three replicates of the central run, leading to 15 sets of experiments, enabling each experimental response to be optimized. The responses were investigated using a Box–Behnken statistical experimental design. The optimization process involves evaluating the response of the statistically designed combinations, estimating the coefficients by fitting the experimental data to the response function, predicting the response of the fitted model, and checking the adequacy of the model. All experiments were performed in standard order to minimize the effects of uncontrolled factors that may introduce a bias in the response. Before starting an optimization procedure, it is important to identify the crucial factors affecting the quality of the derived outcomes. The levels for the variables were chosen on the basis of their minimum and maximum effect on absorbance and also each level has been tested at lower and higher end. The levels of the three factors evaluated in this design are listed in (Table 1). A three factor, three-Level Box–Behnken design was used for the optimization procedure, using the software Design Expert V 7.1.1. All other factors, for example volume and detection wavelength were maintained constant. The quality of the fitted model was expressed by the coefficient of determination *R*^2^, and its statistical significance was checked by an *F*-test (analysis of variance) at the 5% significance level. The statistical significance of the regression coefficients was determined by using the *t*-test (only significant coefficients with *p*-value < 0.05 are included). The optimum processing conditions were obtained by using graphical and numerical analysis based on the criteria of the desirability function and the response surface.

**Table 1:** Levels of tested parameter for Box-Behnken design

**Independent factors**	**Unit**	**Symbol**	**Levels**	
			**Low**	**High**
DDQ	%	A	0.06	0.25
Time	Minutes	B	1	6
Temperature	°C	C	25	37

## Results and discussion

### Design of the proposed assay and strategy for its development

The proposed assay was designed to employ 96-microwell assay plate as the CT reaction was carried out in microwells of the assay plate (200-μl reaction volume) instead of the conventional volumetric flasks (10,000-μl volume). The solutions were dispensed by 8-channel pipette, and the absorbances of the colored CT complex were measured by microwell-plate absorbance reader instead of the conventional spectrophotometer.

In the present study, ATR-Ca was selected based on its therapeutic importance, clinical success, and the expected electron-donating ability. This selection was supported by a previous study made by Darwish IA [24], which demonstrated the excellent electron-donating property of alkali salts of carboxylic acid pharmaceutical compounds. Previous studies involving CT reactions with polyhalo-/polycyanoquinone electron *n*-acceptors revealed that DDQ is one of the most efficient reagents in terms of its reactivity [24,25]. Furthermore, its CT reaction with electron-donating analytes is instantaneous [24,26]. For these reasons, DDQ was used as electron acceptor in the development of the proposed assay. The 96-microwell design of the proposed assay was based on the previous success of Darwish *et al.*[27] in the utility of this design for determination of some other pharmaceuticals.

### Reaction and spectral characteristics

**Figure 2:**
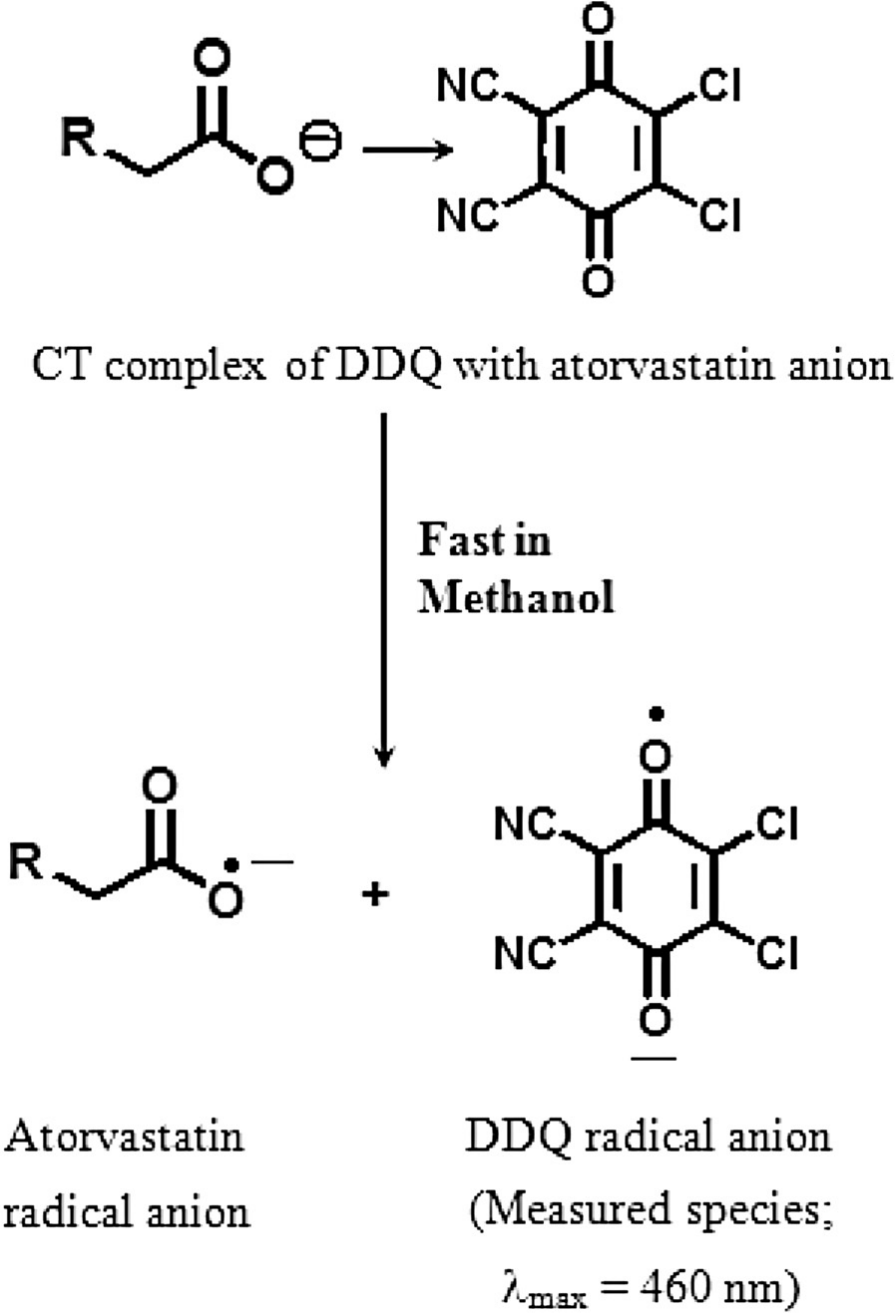
Mechanism of CT reaction of ATR-Ca with DDQ.

**Figure 3:**
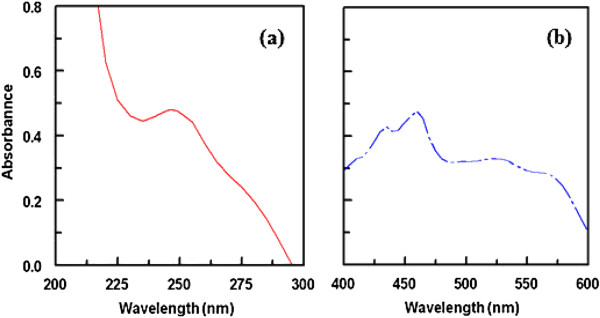
Absorption spectrum of (A) ATR-Ca (125 μg/ml) and (B) reaction product of ATR-Ca (125 μg/ml) with DDQ (0.16%, w/v).

The interaction of ATR-Ca with DDQ was allowed to proceed at room temperature, and the absorption spectrum of the produced chromogen was recorded. ATR-Ca gave red colored chromogen showing absorption maximum at 460 nm (Figure 2 and Figure 3). This band was attributed to the formation of the radical anion DDQ^-^[28], which was probably formed by the dissociation of an original donor-acceptor (D-A) complex:

(1) $$D+A{⇌}_{\mathrm{Complex}}^{\left(\text{D}-A\right)}\overset{\begin{array}{l} \mathrm{polar}\\ \mathrm{solvent} \end{array}}{⇌}{\;}_{\mathrm{radical ions}}^{{D^{+}}_{+}A^{-}}$$

Further support of this assignment was provided by the absorption maxima with those of DDQ radical anion produced by the iodide reduction assay [28]. The dissociation of the (D-A) complex was promoted by the high ionizing power of the polar solvent and the resulting peaks in the absorption spectra of drug-acceptor reaction mixtures were similar to the maxima of the radical anions of the acceptors obtained by the iodide reduction assay [28].

### Response surface optimization

The key parameters most influencing on the new microwell-based spectrophotometric method for the analysis of ATR-Ca, viz DDQ, time and temperature were studied for study. The response was measured in terms of actual factors of absorbance. The results of experimental runs are summarized in (Table 2). Data collected from experimental runs were analysed by using the Design Expert software, version V 7.1.1 and fitted to nonlinear quadratic models for atorvastatin spectrophotometric analysis. The model was validated by analysis of variance (ANOVA). The statistical analysis showed that the model represents the phenomenon quite well and the variation of the response was correctly related to the variation of the factors (Table 3). Student’s *t*-test was used to determine the significance of the regression coefficients of the variables. A p-value below 0.05 indicates the test variable is significant at the 5% level. The results are indicative for good precision and reliability for the experiments carried out. The significance of each coefficient is listed in Table 4. The fitted model equation is:

(2) $$\begin{array}{cc} \text{Absorbance} & =1.63+0.41\text{A}+0.12\text{B}+0.011\text{C}\\ & \;+0.087\text{AB}+0.011\text{AC}+1.00\text{E}-02\text{BC}\\ & \;-0.25\text{AA}-0.043\text{BB}+2.56\text{E}-03\text{CC} \end{array}$$

**Table 2:** The Box–Behnken design matrix of three variables

**Runs**	**Factors**			**Absorbance**
	**DDQ**	**Time**	**Temperature**	
1	0.06	6.00	31.00	0.99
2	0.16	3.50	31.00	1.63
3	0.06	3.50	37.00	0.95
4	0.25	3.50	37.00	1.85
5	0.16	1.00	37.00	1.46
6	0.16	3.50	31.00	1.61
7	0.06	1.00	31.00	0.94
8	0.06	3.50	25.00	0.94
9	0.16	6.00	37.00	1.73
10	0.25	1.00	31.00	1.52
11	0.16	3.50	31.00	1.65
12	0.16	6.00	25.00	1.69
13	0.25	3.50	25.00	1.79
14	0.16	1.00	25.00	1.47
15	0.25	6.00	31.00	1.91

**Table 3:** Analysis of variance of calculated model for atorvastatin absorbance

**Source**		**Absorbance**	
	**Mean squares**	***F***	***p*-value**
Model	0.19	100.37	0.0001
Residual	9.417 × 10^-003^	-	-
*R*^*2*^	0.9945		

**Table 4:** Regression coefficients and their significance in the quadratic model

**Terms**		**Absorbance**
	**Estimate**	***P-value***
Intercept	1.63	< 0.0001
A-DDQ	0.41	< 0.0001
B-Time	0.12	0.0006
C-temp	1.10E-02	0.5148
AB	0.087	0.0102
AC	1.10E-02	0.6188
BC	1.00E-02	0.6643
A^2	−0.25	0.0001
B^2	−4.30E-02	0.1182
C^2	2.56E-03	0.9141

Interpretation of the results has to start from analysis of the response surface and study of the Derringer’s desirability function. The optimum conditions for absorbance were also estimated by use of Derringer’s desirability function [29]

(3) $$\text{D}={\left[d1^{w1}\times d{2^{w}}^{2}\times \ldots \times dn^{\mathrm{wn}}\right]}^{1/n}$$

where wi is the weight of the response, n the number of responses, and di the individual desirability function for each response. In the actual study all *with* values were set equal to 1.

**Figure 4:**
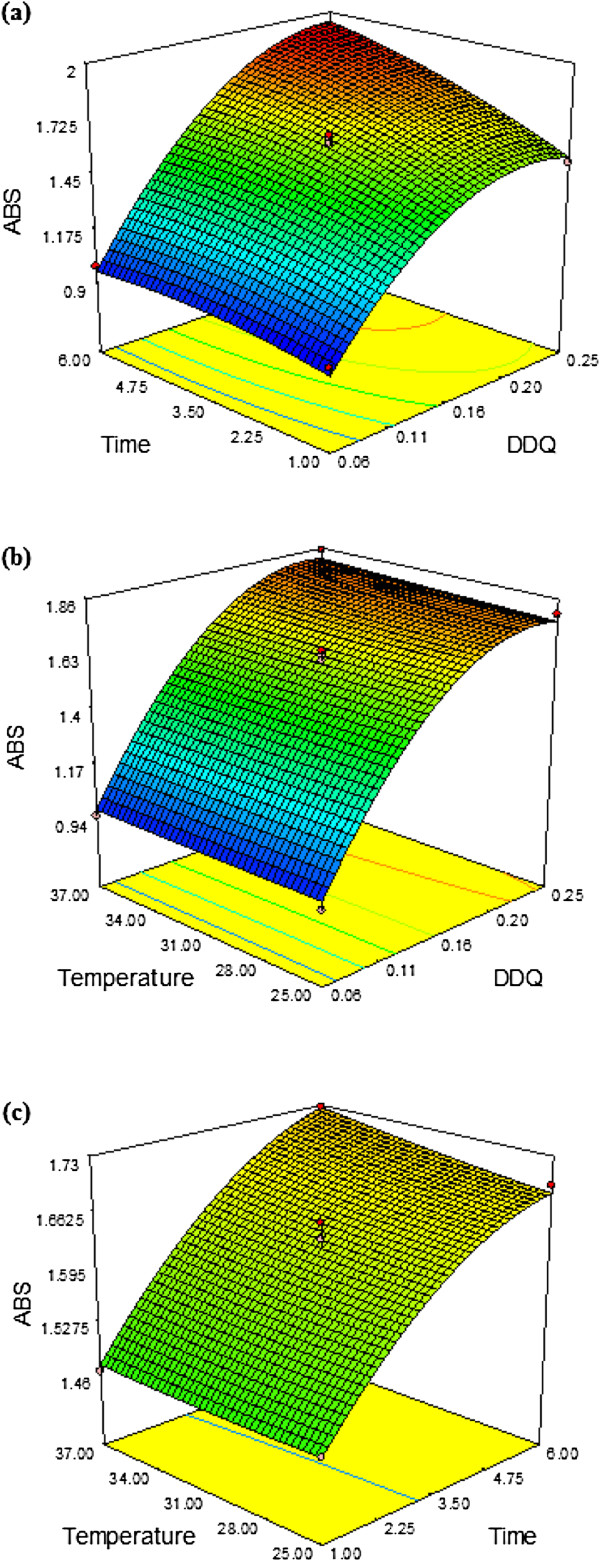
Response-surface graphs representing the effect of DDQ, time and temperature on the responses: (a) Relative effect of DDQ and Time on atorvastatin absorbance at constant temperature (b) Relative effect of temperature and DDQ on atorvastatin absorbance, while keeping time constant (c) Relative effect of temperature and time on absorbance of atorvastatin, while keeping concentration of DDQ constant.

This multiple non linear model resulted in response surface graphs for absorbance (Figure 4a, 4b and 4c). Point prediction tool of the software was used to calculate maximum absorbance. Finally the optimum values for DDQ 0.16% w/v, time 3.5 min and temperature 31°C resulted in absorbance of 1.63. The optimized values of these parameters were validated under similar conditions. An average 1.612 of absorbance was produced under optimized conditions with 99.9% validity. The above results obtained by use of these conditions suggested that the response surface predictions were in good agreement with the experimental results. Therefore, Box–Behnken statistical design was reliable and effective in determining the optimum conditions.

**Figure 5:**
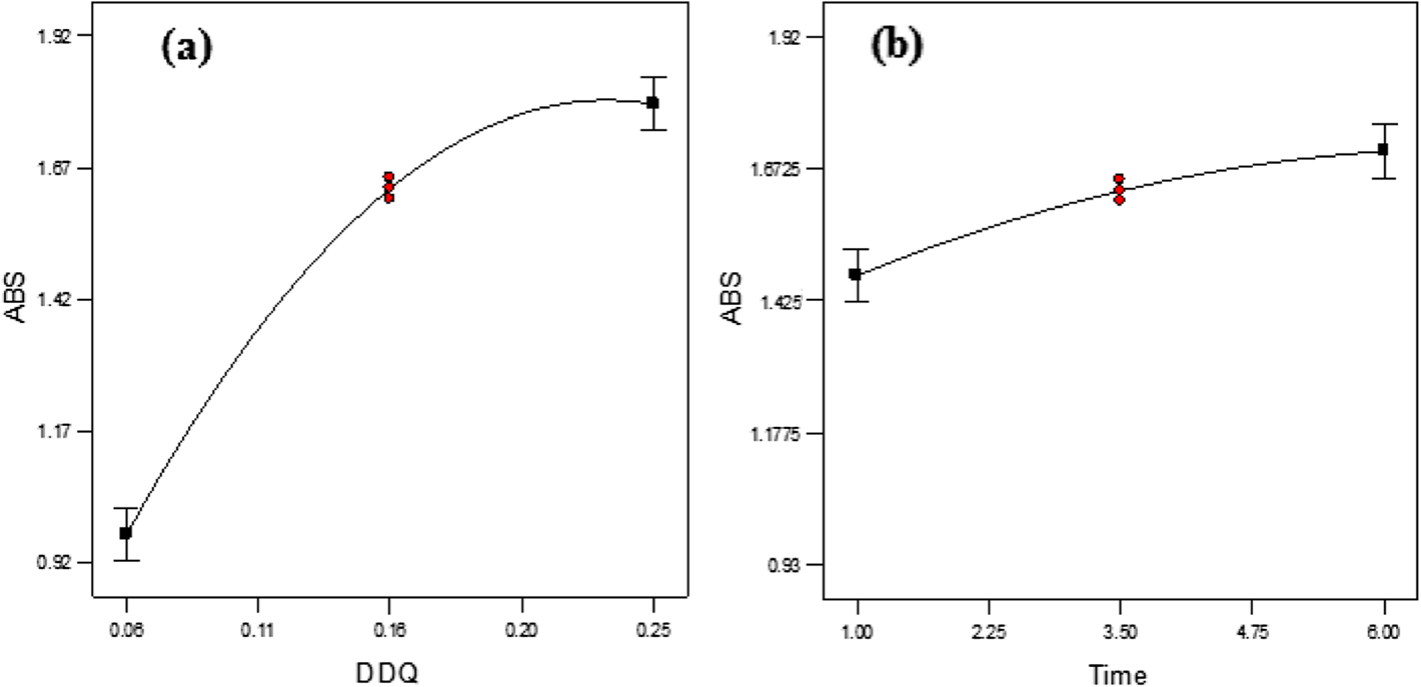
Effect of individual factors DDQ (a) and Time (b) on absorbance by atorvastatin.

Figure 3a shows the effect of time and proportion of DDQ on absorbance at a constant temperature 31°C. It is apparent from the figure that absorbance increased with increasing time and proportion of DDQ. Figure 3b shows the effect of temperature and proportion of DDQ on absorbance of atorvastatin at fixed time of 3.5 min, as the DDQ concentration increases there is significant increase in the absorbance upto a certain limit whereas increase in temperature enhances the absorbance. Figure 3c shows the effect of temperature and time on absorbance of atorvastatin at constant concentration of DDQ at 0.16% w/v however, increase in temperature increases the absorbance and after a certain period of time there is slight decrease in absorbance. Figure 5a and 5b shows effect of individual factors DDQ and Time on the absorbance and as is evident from the figures that both these variables have direct effect on absorbance.

### Validation of the proposed assay under optimized conditions

#### Linearity and sensitivity

Under the above mentioned optimum reaction conditions using response surface methodology, the calibration curve for the analysis of ATR-Ca by the proposed assay was constructed by plotting the absorbances as a function of the corresponding concentrations. The regression equation for the results was derived using the least-squares method. Beer’s law plot (n = 10) was linear with very small intercept (0.0061) and good correlation coefficient (0.9993) in the concentration range of 20 – 200 μg/well (100 μl). The limits of detection (LOD) and quantitation (LOQ) were determined [30] using the formula: LOD or LOQ = κSDa/b, where κ = 3.3 for LOD and 10 for LOQ, SDa is the standard deviation of the intercept, and b is the slope. The LOD and LOQ values were 3.91 and 11.84 μg/well (100 μl), respectively. The quantitative parameters of the proposed assay are given in Table 5.

**Table 5:** Quantitative parameters for the analysis of ATR-Ca by the proposed assay

**Parameter**	**Value**
**Range (μg/well)**^**a**^	**20-200**
Intercept	0.0061
Slope	0.0078
Correlation coefficient	0.9993
LOD (μg/well) ^a^	3.91
LOQ (μg/well) ^a^	11.84

^a^ Each well contained 100 μl of ATR-Ca sample.

#### Accuracy and precision

Accuracy of the proposed assay was assessed by analytical recovery studies. Recovery was determined by the standard addition method. Known amounts of ATR-Ca were added to pre-determined drug-containing pharmaceutical formulation, and then determined by the proposed assay. The mean analytical recovery was calculated using five determinations and was found to be 98.74 ± 0.7% to 100.22 ± 1.24% indicating the accuracy of the proposed assay.

The precisions of the proposed assay were determined on samples of drug solutions at three concentration levels for each drug by analyzing 5 replicates of each sample as a batch in a single assay run. The relative standard deviations (RSD) did not exceed 1.59% (Table 6) proving the high precision of the assay for the routine application in quality control laboratories. This high level of precision was attributed to response surface methodology optimized conditions. And the accuracy of the volumes that have been concomitantly dispensed in the microwells by multi-channel pipettes, and completeness of the reaction the small volume (200 μl).

**Table 6:** Precision and accuracy of the proposed assay at different ATR-Ca concentrations

**Concentration (μg/well)**	**Relative standard deviation**			
	**Intra-day, n=5**		**Inter-day, n=5**	
	**Found (μg/well)**	**% RSD**	**Found (μg/well)**	**% RSD**
80	80.60 ± 1.28	1.59	80.75 ± 0.99	1.24
120	120.5 ± 0.85	0.71	120.34 ± 1.11	0.92
180	181.04 ± 1.93	1.07	180.42 ± 1.37	0.76

#### Application of the proposed assay in the analysis of pharmaceutical formulations

The commercially available pharmaceutical formulations of ATR-Ca were subjected to the analysis by the proposed and reported methods [16] and the obtained results were then statistically compared with each other. The mean percentage recoveries, relative to the labeled amounts, obtained by the proposed assay were 99.24 ± 1.21 and 98.89 ± 1.62% for Lipitor and lipicure–10 tablets, respectively (Table 7). In the t- and F-tests, no significant differences were found between the calculated and theoretical values of both the proposed and the reported assays at 95% confidence level. This indicated similar accuracy and precision in the analysis by the proposed and reported methods.

**Table 7:** Analysis of ATR-Ca in its tablets by the reported and proposed methods

**Tablet**	**Content (% ± SD)**^**a**^		**t-Value**^**b**^	**F-value**^**b**^
	**Proposed method**	**Reported method**		
Lipitor tablets	99.24 ±1.21	99.78±0.68	0.87	3.16
Lipicure–10 tablets	98.89± 1.62	99.10±1.73	0.20	1.14

^a^ Values are mean of five determinations ± SD.

^b^ The tabulated values at 95% confidence limit are 2.78 and 6.39, respectively.

## Conclusion

The present study identified the effect of individual variables on analysis of ATR-Ca by microwell-based spectrophotometric assay using Box-Benkhen response surface methodology. The above results obtained using response surface predictions were in good agreement with the experimental results. Therefore, Box–Behnken statistical design used in determining the optimum experimental conditions such as concentration of DDQ, temperature and time was reliable and effective. Using the optimized conditions, development and validation of microwell based UV-visible spectrophotometric assay (200-μl reaction volume) instead of the conventional volumetric flasks (10,000-μl volume) was performed for the determination of ATR-Ca based on its CT reaction with DDQ reagent. The absorbances were measured by microwell-plate reader instead of the conventional spectrophotometer. The assay described herein offered the following advantages:

Reduction in the consumption of organic solvents (environmentally friendly "Green" approach) in the CT-based UV-visible spectrophotometric analysis, accordingly reduction in the exposures of the analysts to the toxic effects of organic solvents.

Reduction in the analysis cost by 50-folds which can be reflected on the price for the finished dosage forms, thus it can reduce the expenses for the medications.

Providing a high throughput analytical methodology that can facilitate the processing of large number of samples in a relatively short time. This property was attributed to the use of multi-channel pipettes for efficient dispensing of the solutions, carrying out the analytical reaction in 96-well plates (as reaction vessels), and measuring the color signals in the 96 wells at ~ 30 seconds by the plate reader.

The advantages of the proposed assay and in addition to automation could be reached by application of Flow Injection Analysis.

Although the proposed assay was developed and validated for ATR-Ca, however, it is also anticipated that the same methodology could be used for essentially any analyte that can exhibit CT reaction.

## Abbreviations

CT: Charge-transfer; ATR-Ca: Atorvastatin calcium; DDQ: 2,3-dichloro-5,6-dicyano-1,4-benzoquinone; HMG-CoA: 3-hydroxy-3-methylglutaryl-coenzyme A; LDL: Low-density lipoprotein; LOD: Limit of detection; LOQ: Limit of quantification; SD: Standard deviation; RSD: Relative standard deviation; ABS: Absorbance.

## Competing interests

The authors declare that they have no competing interests.

## Authors’ contributions

TW and SZ contributed in the design of the study and conducting the optimization of the assay conditions and validation. NK and AA contributed in the assay design and conducted the assay validation and analysis of dosage forms. ID designed the study, participated in the results discussion and prepared the manuscript. All authors have read and approved the final manuscript.
